# PCR Detection of Mimivirus 

**DOI:** 10.3201/eid2306.161896

**Published:** 2017-06

**Authors:** Didier Raoult, Anthony Levasseur, Bernard La Scola

**Affiliations:** Aix-Marseille Université, Marseille, France

**Keywords:** mimivirus, pneumonia, false negative PCR, viruses, primer/probe mismatch

The article by Zhang et al. (*1*) reporting the failure to detect mimivirus (Acanthamoeba polyphaga mimivirus) in patients with acute respiratory symptoms was flawed in 2 ways. First, a single mimivirus was detected by PCR but was dismissed by the authors as colonization. Second, and of greater concern, the PCR used by the authors would not detect most known mimiviruses because of primer/probe mismatches. In fact, PCRs used in most mimivirus prevalence studies would fail to detect most mimiviruses, given the extensive genome sequence diversity of these viruses (*2*). 

We evaluated the PCR primers/probes used by Zhang et al. (*1*) for sequence similarity with 51 mimivirus genomes available in the public domain since March 2016, the date on which the article by Zhang et al. was submitted (*3*). Our in silico analysis found that none of the 25 genomes available from lineage A and 7 genomes from lineage B were complementary with their primers and probes, and only 5 of 19 lineage C genomes showed sufficient sequence similarity to permit successful amplification (Table). Overall, the PCR used by these authors would be predicted to detect only ≈26% of lineage C mimiviruses and <10% of published mimivirus genomes overall.

**Table:** Mimivirus genomes in the public domain screened to determine whether the genomes were complementary with their primers/probes*

Lineage A (GenBank accession no.)	Lineage B (GenBank accession no.)	Lineage C (GenBank accession no.)
Mimivirus fauteuil (CWKE00000000)	Moumouvirus ochan (CXPE00000000)	Megavirus shan (CWIL00000000)
Mimivirus longchamps (CXOV00000000)	Moumouvirus istres (PRJEB9189)	Megavirus montpellier3 (CWIA00000000)
Mimivirus marais (PRJEB9199)	Moumouvirus battle49 (CXOS00000000)	Megavirus mont1 (CWIO00000000)
Mimivirus lactours (CXOL00000000)	Moumouvirus saoudian (CXOQ00000000)	Megavirus avenue9 (CWHN00000000)
Mimivirus PointeRouge1 (CXOW00000000)	Moumouvirus boug1 (PRJEB9190)	Megavirus balcon (PRJEB9194)
Mimivirus PointeRouge2 (CXOU00000000)	Moumouvirus goulette (KC008572)	Megavirus ursino (CWJY00000000)
Mimivirus T3 (CXOR00000000)	Moumouvirus Monve (JN885994–JN886001)	Megavirus T1 (CWIB00000000)
Mimivirus huitre A06 (CWJT00000000)		Megavirus battle43 (CWHV00000000)
Mimivirus amazonia (CWHO00000000)		Megavirus T4 (CWJR00000000)
Mimivirus battle7 (CWJW00000000)		Megavirus J3 (CWIN00000000)
Mimivirus battle19 (CWJX00000000)		Megavirus T6 (CWJQ00000000)
Mimivirus battle27 (CWJS00000000)		**Megavirus chilensis (NC_016072)**
Mimivirus battle57 (CWJZ00000000)		**Megavirus courdo11 (JX975216)**
Mimivirus battle66 (CWKA00000000)		**Megavirus lba† (JX885207)**
Mimivirus battle83 (CWKG00000000)		Powai lake megavirus (KU877344)
Mimivirus battle86 (CWKD00000000)		**Megavirus terra1 (KF527229)**
Mimivirus T2 (CXOT00000000)		Megavirus courdo5 (CWID00000000)
Mimivirus battle6 (CWJU00000000)		Megavirus bus (CWIQ00000000)
Mimivirus dakar4 (CWKF00000000)		**Megavirus courdo7 (JN885990–JN885993)**
Mimivirus (AY653733)		
Mimivirus Bombay (KU761889)		
Mimivirus terra 2 (PRJNA240235)		
Samba virus (KF959826)		
Mimivirus lentille (AFYC00000000)		

Mimivirus urine†

*Bold indicates genomes that would be predicted to be successfully amplified using the PCR primers and probes in the article by Zhang et al. (*1*). †Mimiviruses isolated from human samples.

As proposed by Ngounga et al. (*4*), the great variability across the 3 lineages of mimivirus genomes requires the design of lineage-specific primers to improve PCR sensitivity (*4*). Until PCRs are improved, mimiviruses might be best detected by metagenomic approaches that have recently seen success in terms of mimivirus detection. For example, mimivirus-like reads have been identified by metagenomics in 1 (8%) of 12 fecal samples from children with acute diarrhea (*5*); 5 (27%) of 18 prostatic secretion samples from persons with prostatitis (*6*); all DNA libraries tested from the plasma of patients with hepatitis (*7*); 0.0018% of total viral reads from nasopharyngeal samples from 210 patients with respiratory tract symptoms (*8*); and all tested fecal, mid-vaginal, buccal mucosal, and retroauricular crease samples, with a predominance of miniviruses in fecal samples reaching ≈33% of the total viral reads (*9*). 

In metagenomics approaches, the number of reads detected and the distribution along the whole viral genome are essential parameters for viral detection. In practice, the search for mimivirus is complicated by the great genetic variability of the virus and the restricted availability of mimivirus culture systems to a few research laboratories (*10*). The deficiencies we found in the report by Zhang et al. highlight the need for carefully designed epidemiologic studies using sensitive laboratory test methods to accurately assess mimivirus prevalence and the potential role of mimivirus in human disease.
